# Supplementary material for: Domain-incremental white blood cell classification with privacy-aware continual learning
Source: Sci Rep. 2025 Jul 15;15:25468. doi: 10.1038/s41598-025-08024-z (PMC12264020; doi:10.1038/s41598-025-08024-z)
Supplement: Supplementary file 1 — Supplementary Information. [file 41598_2025_8024_MOESM1_ESM.pdf]

Supplementary file:
Domain-incremental White Blood Cell Classification with Privacy-aware Continual Learning

Pratibha Kumari[1,+,*], Afshin Bozorgpour[1,+], Daniel Reisenbüchler[1], Edgar Jost[4], Martina Crysandt[4], Christian Matek[3], and Dorit Merhof[1,2]

[1]University of Regensburg, 93053, Germany
[2]Fraunhofer Institute for Digital Medicine MEVIS, Bremen, Germany
[3]University Hospital Erlangen, Erlangen, Germany
[4]Department of Hematology, Oncology, Hemostaseology and Stem Cell Transplantation, University Hospital RWTH Aachen, Aachen, Germany

[*]corresponding author: pratibha.kumari@ur.de
[+]these authors contributed equally to this work

1 WBC Classes Selection

Here, we discuss the basis of selecting classes for all the experiments. In the domain shift scenario of continual learning (CL), the classification classes should be the same across episodes. We find six common White Blood Cell (WBC) classes across the four considered datasets, viz., PBC[1], LMU[2], MLL[3], and UKA. We take out 50 samples from each class and then analyze the available training samples across datasets. The training samples for the six classes including MON, EOS, LYT, BAS, NGB, and EBO, across PBC, LMU, MLL, and UKA are mentioned in Table 1. We can see that BAS class has quite low samples (29) compared to MON (1739), EOS (374), and LYT (3887) in LMU dataset. We observe the same in MLL and UKA datasets and thus we discard BAS class. Further, NGB class has only 59 samples compared to MON (1739), EOS (374), and LYT (3887) in LMU dataset. Similarly, EBO class offers only 28 samples as compared to MON (1739), EOS (374), and LYT (3887) in LMU dataset. Hence, we discard NGB and EBO too from considered class sets.

Table 1. Details of datasets used in experiments

Dataset	Center	Source	Considered classes			Discarded classes		
			MON	EOS	LYT	BAS	NGB	EBO
PBC[1]	$C1$	Blood smears	1370	3067	1164	1168	3279	1501
LMU[2]	$C2$	Blood smears	1739	374	3887	**29**	**59**	**28**
MLL[3]	$C3$	Bone-marrow	3990	5833	6000	**391**	9918	27345
UKA	$C4$	Bone-marrow	239	124	229	**27**	1272	236

2 Data Distribution Analysis

Domain-incremental WBC classification requires handling diverse and complex data distributions arising from multiple sources, imaging conditions, and variations in cell morphology leading to shifts in latent representations that challenge model generalization and robustness. Accurately modeling these distributions is essential for effective CL across multiple datasets within a single model. Traditional parametric approaches, such as Gaussian Mixture Models (GMM), assume a fixed number of components, limiting their ability to fully capture the complexity of heterogeneous WBC data, particularly in high-dimensional feature spaces. In contrast, non-parametric methods like Kernel Density Estimation (KDE) offer greater flexibility by dynamically adapting to the underlying data distribution without restrictive assumptions. Table 2 presents a log-likelihood-based comparison across different backbones (ResNet50, RetCCL, CTransPath, UNI) and datasets in Seq. 1. The results consistently demonstrate KDE's superior performance over GMM across all backbones and datasets. For the ResNet50 backbone (2048-dimensional), KDE maintains a significant advantage, achieving a log-likelihood of 1.8669 compared to 0.4194 in the simplest experiment (on a single dataset) and 0.3726 vs. 0.0761 when incorporating all datasets (PBC>LMU>MLL>UKA).

A similar trend is observed for RetCCL (2048-dimensional), where KDE achieves higher log-likelihood values (e.g., 1.9757 vs. 0.4982 for PBC). Even in lower-dimensional feature spaces, such as CTransPath (768-dimensional) and UNI (1024-dimensional), KDE consistently outperforms GMM, highlighting its robustness across different backbones. These findings reinforce KDE's superior ability to capture complex, multi-modal distributions, making it a more suitable approach for modeling the diverse nature of WBC data.

Table 2. Comparison of average log-likelihood values for KDE and GMM as datasets are incrementally added under different backbone architectures.

**Datasets in Seq. 1 →**	**PBC**		**PBC>LMU**		**PBC>LMU>MLL**		**PBC>LMU>MLL>UKA**	
**Backbone (*Dim.*) ↓**	*GMM*	*KDE*	*GMM*	*KDE*	*GMM*	*KDE*	*GMM*	*KDE*
ResNet50 (*2048*)	0.4194	1.8669	0.1954	0.9046	0.0797	0.3778	0.0761	0.3726
RetCCL (*2048*)	0.4982	1.9757	0.2402	0.9944	0.1016	0.4373	0.0994	0.4355
CTransPath (*768*)	0.1786	0.4247	0.0853	0.2195	0.0352	0.0982	0.0342	0.0988
UNI (*1024*)	-0.0492	0.1320	-0.0355	0.0837	-0.0201	0.0428	-0.0221	0.0455
**Average**	0.2617	1.0998	0.1213	0.5505	0.0491	0.2390	0.0469	0.2381

3 Generated Latent Data Analysis

To quantitatively assess how well the generator preserves past domain characteristics, we evaluate similarity using Cosine Similarity [4], Euclidean Distance [4], Fréchet Inception Distance (FID) [5], and Maximum Mean Discrepancy (MMD) [6]. Cosine Similarity measures the alignment between generated and real feature vectors, where higher values indicate better preservation of feature relationships. Euclidean Distance captures the magnitude of deviation between generated and original data, with lower values signifying greater similarity. FID assesses the quality of generated samples by comparing the statistical properties of their feature distributions with those of real data, where a lower FID score indicates better alignment. MMD provides a statistical measure of distributional closeness, quantifying the difference between real and generated feature distributions using a kernel-based approach. To compute above-mentioned metrics at t^{th} training session, we consider β actual samples from each past datasets $(0\ldots(t-1))$ and concatenate them to get actual past latent vectors L_{real}^{t-1} and then generate $(t-1)\times\beta$ latent vectors as L_{gen}^{t-1}, where β is min $\left(|D_{\text{train}}^{1}|, |D_{\text{train}}^{2}|, \ldots, |D_{\text{train}}^{t-1}|\right)$. For each metric, we compute the average value across all pairs of generated L_{gen}^{t-1} and real L_{real}^{t-1} samples (point-wise). After completing all the training sessions in a given sequence, we average the metrics (cosine, euclidean, FID, and MMD) across episodes. Finally, we get average statistics over different sequences and report the mean with standard deviation in Table 3. For all backbones, we observe that the KDE-based generator outperforms the GMM-based generator, except for the UNI backbone, where the GMM generator slightly surpasses the KDE generator. Overall, the KDE-based generator achieve higher Cosine Similarity and lower Euclidean Distance, along with improved FID and MMD scores. These findings suggest that the KDE-based generator produces latent representations that better align with past domain distributions, providing a more effective approach for simulating previous domains.

Table 3. Comparison of original and generated latent features using cosine similarity, euclidean distance, FID, and MMD for KDE-based and GMM-based generators across different backbones.

**Backbone**	**Generator**	**Cosine↑**	**Euclidean ↓**	**FID ↓**	**MMD ↓**
ResNet50	KDE	**93.1**$_{\pm 1.5}$	**4.35**$_{\pm 0.35}$	**22.67**$_{\pm 2.16}$	1.23e-02$_{\pm 6.03e-03}$
	GMM	78.3$_{\pm 3.0}$	8.44$_{\pm 0.33}$	28.95$_{\pm 3.69}$	**1.12e-03**$_{\pm 6.47e-04}$
RetCCL	KDE	**96.0**$_{\pm 0.6}$	**0.55**$_{\pm 0.04}$	**0.36**$_{\pm 0.03}$	**3.21e-02**$_{\pm 6.88e-03}$
	GMM	38.6$_{\pm 1.9}$	4.59$_{\pm 0.01}$	19.00$_{\pm 0.19}$	1.24e-01$_{\pm 1.33e-02}$
CTransPath	KDE	**89.9**$_{\pm 0.2}$	**1.58**$_{\pm 0.02}$	**1.57**$_{\pm 0.04}$	**2.44e-02**$_{\pm 1.72e-02}$
	GMM	72.9$_{\pm 0.7}$	3.04$_{\pm 0.05}$	6.74$_{\pm 0.12}$	3.98e-02$_{\pm 2.06e-02}$
UNI	KDE	86.6$_{\pm 0.7}$	20.16$_{\pm 0.37}$	210.03$_{\pm 15.87}$	**1.12e-03**$_{\pm 6.47e-04}$
	GMM	**87.0**$_{\pm 2.3}$	**19.41**$_{\pm 1.95}$	**187.91**$_{\pm 42.20}$	**1.12e-03**$_{\pm 6.47e-04}$

References

1. Acevedo, A. *et al.* A dataset of microscopic peripheral blood cell images for development of automatic recognition systems. *Data brief* **30** (2020).

2. Matek, C., Schwarz, S., Marr, C. & Spiekermann, K. A single-cell morphological dataset of leukocytes from aml patients and non-malignant controls (aml-cytomorphology_lmu). *The Cancer Imaging Arch. (TCIA)[Internet]* (2019).

3. Matek, C., Krappe, S., Münzenmayer, C., Haferlach, T. & Marr, C. An expert-annotated dataset of bone marrow cytology in hematologic malignancies. *The Cancer Imaging Arch.* (2021).

4. Bishop, C. M. & Nasrabadi, N. M. *Pattern recognition and machine learning*, vol. 4 (Springer, 2006).

5. Heusel, M., Ramsauer, H., Unterthiner, T., Nessler, B. & Hochreiter, S. Gans trained by a two time-scale update rule converge to a local nash equilibrium. *Adv. neural information processing systems* **30** (2017).

6. Smola, A. J., Gretton, A. & Borgwardt, K. Maximum mean discrepancy. In *13th international conference, ICONIP*, vol. 6 (2006).
